# Serum and Urinary Neutrophil Gelatinase-Associated Lipocalin Are Not Associated With Serum Redox Parameters in Amateur Athletes After an Ultramarathon

**DOI:** 10.3389/fphys.2022.811514

**Published:** 2022-03-17

**Authors:** Adriano César Carneiro Loureiro, Gabriella Fontenele Nocrato, André Luis Lima Correia, Robson Salviano de Matos, Júlio César Chaves Nunes Filho, Elisabeth De Francesco Daher, Flávio Henrique Macedo Pinto, Ariclécio Cunha de Oliveira, Vania Marilande Ceccatto, Rodrigo Soares Fortunato, Denise Pires de Carvalho

**Affiliations:** ^1^Superior Institute of Biomedical Sciences, Ceará State University, Fortaleza, Brazil; ^2^Department of Clinical Medicine at the Federal University of Ceará, Ceará Federal University, Fortaleza, Brazil; ^3^Treatment and Integration Center of the Being, Fortaleza, Brazil; ^4^Carlos Chagas Filho Biophysics Institute, Rio de Janeiro Federal University, Rio de Janeiro, Brazil

**Keywords:** ultramarathon, oxidative stress, NGAL, physiological markers, amateur athletes

## Abstract

**Objective:**

To evaluate the relationship between oxidative stress and NGAL levels in blood and urine of amateur athletes after participating in a 100 km ultramarathon.

**Methodology:**

The sample was composed of seven athletes, submitted to anthropometric assessment, cardiopulmonary exercise test, collection of urine and blood, measurement of body weight. The rate of perceived exertion (RPE), competition duration, heart rate (HR), energy expenditure and oxygen consumption (V’O_2_”) were also measured during the event. The energy consumption during the race was verified at its end. The analyses were based on the means (M) and respective standard deviations (SD), with statistical significance set at 5% (*p* < 0.05). Paired *t*-test was used for comparison between the periods before and after the competition, and Pearson’s correlation coefficient was used to measure the linear correlation between quantitative variables.

**Results:**

Body mass index (BMI) of the sample was 25.75 kg/m^2^ ± 3.20, body fat percentage 18.54% ± 4.35% and V’O_2_”_max_ 48.87% ± 4.78. Glucose, cortisol, and neutrophil gelatinase-associated lipocalin (NGAL) (*p* < 0.01) as well as glutathione peroxidase (GPx) active were higher after the race when compared to basal values. Moreover, lactate, creatinine, microalbuminuria, and glomerular filtration rate (GFR) (*p* < 0.001) were also higher after the race. After the competition, there was a significant correlation only between serum NGAL and creatinine, which was classified as strong and positive (r: 0.77; *p* < 0.05). There was a significant reduction (*p* < 0.05) of body weight after the event (72.40 kg ± 9.78) compared to before it (73.98 kg ± 10.25). In addition, we found an increase of RPE (*p* < 0.001) after the race. The competition lasted 820.60 min (±117.00), with a 127.85 bpm (±12.02) HR, a 2209.72 kcal ± 951.97 energy consumption, 7837.16 kcal ± 195.71 energy expenditure, and 28.78 ml/kg/min^–1^ (±4.66) relative V’O_2_”_max_.

**Conclusion:**

The lack of correlation between oxidative stress biomarkers and serum and urine NGAL suggests that NGAL is more sensitive to inflammatory processes than to ROS levels.

## Introduction

Ultramarathons are foot races that cover any distance longer than 42 km and are associated with several physiological alterations, including disturbances in redox balance and kidney function (Jastrzebski et al., 2016; Shin et al., 2016). Although the majority of studies have indicated that these alterations are short-lived, it is yet to be established whether they represent adaptive or pathological processes, particularly in amateur athletes (Knechtle and Nikolaidis, 2018; Longman et al., 2018; Tirabassi et al., 2018).

Redox balance is defined as the relationship between the pro-oxidant system and the antioxidant defense capacity. Excessive production of reactive oxygen species (ROS) and/or deficient antioxidant capacity, leading to disruption of redox signaling and control and/or to molecular damage, are signs of oxidative stress (Forman et al., 2015). Studies show that physiological levels of ROS are essential for physiological functions, while excessive amounts are related to inflammation and cell apoptosis, hindering the contractile capacity of skeletal muscles (Liochev, 2013; Tanaka, 2017). Conflicting results regarding effects of exercise on redox balance during ultramarathons have been found.

It is known that increased blood flow to the muscles during exercise is accompanied by a reduction in renal blood flow (Hodgson et al., 2017). Initially, the kidneys are able to maintain glomerular filtration and renal plasma flow through the mechanism of self-regulation. It occurs due to nitric oxide (NO) production in the afferent arteriole, which reduces vasoconstriction and leads to an increase in the glomerular pressure. However, prolonged reduction in renal perfusion can be associated with acute kidney injury (AKI) due to the excessive production of NO, favoring the formation of ROS, which in turn can induce an inflammatory process, increasing the risk of kidney damage (Kao et al., 2015; Schrezenmeier et al., 2017).

Studies indicate that kidney inflammation is associated with the formation of lipocalin associated with neutrophil gelatinase (NGAL), a glycoprotein that is part of the innate immune system (Choi et al., 2017; Wolyniec et al., 2018). It has been suggested that the existence of an inflammatory process in the kidneys can compromise the reabsorption of NGAL in the proximal tubule, a metabolically active site during exercise, increasing its levels in the urine (uNGAL). In turn, the reabsorption of NGAL increases its concentration in the blood (sNGAL), being released into the systemic circulation at the sites of inflammation (Liu et al., 2015; Eilenberg et al., 2016).

Therefore, NGAL seems to be a sensitive and early marker in response to renal stress, since it is rapidly expressed in this organ after inflammation and ischemia, making it one of the most promising biomarkers for the early diagnosis of AKI (Hisamichi et al., 2017). In particular, AKI occurs in about 80% of ultramarathon runners, mostly at mild levels, with a complete recover within a few days. However, it can progress to chronic kidney disease (CKD), which can negatively affect several systems in the body (McCullough et al., 2011; Hou et al., 2015; Lipman et al., 2016).

Despite the increasing popularity of ultramarathons, there are few studies investigating the physiological changes caused by this type of competition, especially those related to redox balance, renal function, and NGAL regulation. In this context, a better understanding of the dynamics of these biomarkers during ultramarathons can be useful in the search for new biomarkers in the exercise physiology context.

Thus, the aim of this study was to evaluate the relationship between oxidative stress and blood and urine levels of NGAL of amateur athletes before and after a 100 km ultramarathon. Moreover, the correlation between oxidative stress and NGAL with other parameters, such as anthropometric variables, maximum oxygen uptake (V’O_2_”), state of hydration and evolution of fatigue, were also evaluated. Our hypothesis was that oxidative stress biomarkers are related to NGAL production.

## Materials and Methods

### Participants

**FIGURE 1:**
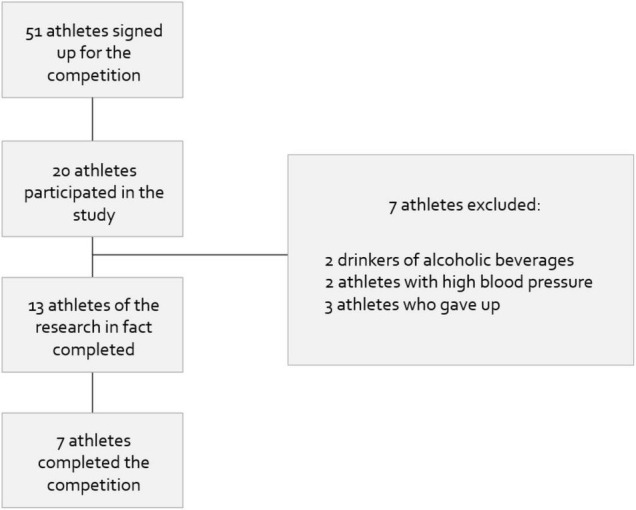
Flow of participants during the survey.

We recruited male amateur athletes between the ages of 21 and 60 years old, with minimum experience of 6 months in long-distance races, who took part in a 100 km ultramarathon on a route between the cities of Fortaleza and Canindé, located in the state of Ceará, Brazil. They were very physically active subjects, meaning that they used to performed seven or more sessions a week of any combination of walking and moderate or vigorous intensity activities, in sessions lasting longer than or equal to 30 min. This information was obtained by the International Physical Activity Questionnaire (IPAQ). We excluded smokers, drinkers of alcoholic beverages, those with any type of pathology, those taking any type of medication, including non-steroidal anti-inflammatory drugs (NSAI), and/or nutritional supplementation during the month preceding the event or who consumed any oxidant or antioxidant during the event. Those individuals who did not finish the race or decided not to take part in the study were also excluded. Figure 1 shows the flow of participants through the trial.

We used a convenience sample consisting of seven individuals, since this sampling method has sufficient statistical power. The experimental design was in line with the rules of Resolution 466/2012 issued by the Brazilian National Health Council. All participants gave written informed consent. The study was approved by the Ethics Committee of Ceará State University (CEP/UECE) under number 3.454.557. The anthropometric evaluations and cardiopulmonary exercise test were carried out in August 2019. The other collections were carried out in September 2019, when the competition, took place.

### Experimental Design

Anthropometric evaluation and cardiopulmonary exercise test were performed one month before the race. These procedures took place in the morning, by appointment, with an average duration of 30 min per participant. Both procedures were performed on 6 athletes on the first day and on 7 athletes on the second day, since they were not feasible in a single day.

The biological material (urine and blood) was collected 1 week before the race and immediately after it. Immediately before and after the race, body weight and rate of perceived exertion (RPE) were measured. Race duration, energy expenditure and average heart rate (using a Polar M200 monitor) were measured during the competition. Immediately after the competition, we calculated the percentage of maximum heart rate (HR_max_), relative maximal oxygen (ml/kg/min^–1^) consumption percentage (%V’O_2_^”^_max_), and absolute V’O_2_^”^_max_ (l/min). At the end of the race, each athlete delivered to the nutritionists a list informing the types and the amounts of food consumed during the ultramarathon.

### Anthropometric Evaluation

Weight was measured on a digital scale with a maximum capacity of 150 kilos (Sanny, Brazil). Height was assessed with a portable stadiometer (ES2060—Sanny, Brazil), with a maximum capacity of 220 cm and a minimum of 50 cm. The body mass index was calculated as weight/height (BMI = kg/m^2^). Since there is no specific BMI for athletes, the result was classified according to the criteria proposed by the World Health Organization for the age group between 20 and 59 years.

For evaluation of body fat percentage, we used the 7-site skinfold protocol, using a Sanny adipometer with sensitivity of 0.1 mm. The results were classified according to the International Association of Athletics Federations [IAAF] (Burke, 2019).

### Cardiopulmonary Exercise Test

HR and blood pressure (BP) were measured using a digital automatic arm pressure apparatus (G-Tech) with the participants at rest, before starting the cardiopulmonary exercise test. Next, a mask with a sensor was attached to the participant’s face to detect expiratory gases, which were recorded by a gas analyzer (Ibramed) to determine the relative V’O_2_”, BP and HR.

The individuals were warmed up on a treadmill (KT-S ATL—Inbrasport), at a walking/running pace, and then the ramp protocol was applied, in which the increment of workload was 1 km/h/min^–1^. The test had an initial speed of 8 km/h (kilometers per hour), with no inclination, and variable final speed, as the criterion for test interruption was participant’s exhaustion. At the end of the test, individuals were monitored for adverse symptoms until HR and BP returned to normal levels. At the time of testing, the temperature and humidity of the ambient air were, respectively, around 22°C and 60%.

### Urine and Blood Sampling

Urine and blood samples were collected on the day before the race, between 6 and 7 a.m., due to the relationship between cortisol and the circadian cycle, with the subjects at rest and after 12 h fasting. Blood and urine samples were also collected immediately after the end of the race. All urine samples were collected in a sterile flask, transferred to the collection tube, and stored in a refrigerator at a temperature between 2 and 8°C.

Blood samples were obtained from a forearm vein of each participant and placed in heparinized Vacutainers. Immediately after collection, with the exception NGAL, these samples were centrifuged (15 min, 1,000 × g) at room temperature, and separated into serum and plasma samples. In serum samples, the concentrations of glucose, creatinine, cortisol and glutathione peroxidase (GPx) activity were analyzed, while in the plasma samples, the concentrations of lactate, 8-isoprostane and NGAL were measured.

All urine samples were analyzed the day after collection. For this reason, they were stored at temperature between 2 and 8°C, as recommended for analyses carried out within 7 days after collection. Glucose, creatinine, cortisol, lactate, and NGAL samples were stored in a freezer at a temperature of −20°C until analysis, which took place during the days following the collection. This storage followed the protocol established by the manufacturer, which states that at these temperatures the samples can be used up to 30 days after collection. In turn, the GPx and 8-isoprostane samples were stored in a freezer at −80°C until analysis (Diagnostic Labtest, 2019).

#### Biochemical and Hormonal Measurements

The glomerular filtration rate (TFG) was calculated using the Cockroft-Gault equation (Cockcroft and Gault, 1976; Hari et al., 2014). Microalbuminuria analyses were performed using the turbidimetry method, which determines the concentration of particles present in a solution by dispersing light (Van et al., 2014). The data obtained from urine samples were compared to the following reference values: TFG (>60 ml/min/1.73 m^2^) and microalbuminuria (<20 mg/L) (Diagnostic Labtest, 2019).

In the serum samples, glucose concentrations were measured by spectrophotometry (Beckman Coulter, United States); creatinine by colorimetric testing (Beckman Coulter, United States) and cortisol by chemiluminescence (Boditech, South Korea). In plasma samples, the lactate levels were measured using a colorimetric test (enzymatic lactate). All analytic techniques followed the protocols provided by specific kit for each biomarker proposed by the respective manufacturer.

The data obtained from blood samples were compared to the following reference values: glucose (60–99 mg/dL—fasting for 8–12 h); creatinine (0.7–1.2 mg/dL); cortisol (6.7–22.6 μg/dL—around 8 a.m.); and lactate (4.5–19.8 mg/dL) (Diagnostic Labtest, 2019).

#### Neutrophil Gelatinase-Associated Lipocalin Measurement

After collection, urine and blood samples for NGAL analysis were centrifuged (4108.65 × g for 10 min) and the supernatant was collected for plasma analysis. After aliquoting, the samples were kept at −20°C.

NGAL was analyzed in serum and urine following the protocol provided in the specific kit for each antibody, proposed by the manufacturer (R&D Systems: Human Lipocalin-2\NGAL, cat DY1757, DuoSet ELISA Ancillary Reagent Kit, cat DY008). The values considered normal for uNGAL and sNGAL were, respectively, below 20 ng/ml and below 70 ng/ml, according to the literature (Pajek et al., 2014; Ho, 2015). Recently, researchers have proposed that subclinical elevations of NGAL, in plasma and urine, in relation to the ARF parameter, may indicate renal stress or mild injury with the possibility of subsequent impairment of function (Nejat et al., 2012; Emlet et al., 2017).

#### Oxidative Stress Biomarkers

Glutathione peroxidase (GPX) activity was measured in the serum by a colorimetric assay kit (GPX Assay kit, Cayman Chemical Company, United States), following the manufacturer’s instructions. 8-Isoprostane levels were measured in the plasma by a colorimetric assay kit (8-Isoprostane Assay kit, Cayman Chemical Company, United States), following the manufacturer’s instructions.

### Body Hydration Index

The body hydration index was measured by the percentage of change in body weight (% of water loss) using the formula: (final weight – initial weight) × 100/initial weight. The values of initial and final weight corresponded, respectively, to the pre and post-competition periods (Pinto et al., 2014). Body weighing took place with participants wearing light clothes, using a digital scale with maximum capacity of 150 kilos (Sanny).

The percentage of variation in body weight, was used to classify the degree of dehydration: up to −1% indicates good hydration; −1 to −3% minimal dehydration; −3 to −5% significant dehydration and under −5% severe dehydration (Godois, 2014).

In this study, the participants were instructed not to drink liquids until checking the body weight before and after weighing. During the competition, water intake occurred *ad libitum*, in order to reproduce the individual’s hydration habits.

### Rate of Perceived Exertion

The rate of perceived exertion (RPE) was used to monitor the internal load, defined by each individual according to the effort sensation (Greig et al., 2006). The perception was evaluated using a scale from 6 to 19, as proposed by Borg (1998). The data were collected within an interval of up to 30 min after the competition, to assure the correct analysis.

### Variables Measured During the Competition

Duration, energy expenditure and average HR of the competition were measured by a frequency meter (Polar, m200). The provided data were analyzed by the Polar Flow app platform.

Energy expenditure was measured according to the protocol indicated by the manufacturer of the Polar m200, based on the following variables: weight, height, sex, age, heart rate, training intensity, and oxygen consumption.

The HR_max_ from the cardiopulmonary exercise test and the HR_mean_ ascertained during the competition were used to calculate the R_max_ percentage (%HR_max_) during the race. This metric was necessary to calculate the relative V’O_2_^”^_max_ percentage (%V’O_2_^”^_max_) during the race, according to the formula of Mcardle et al. (1978): %V’O_2_^”^_max_ = (1.388 × %HR_max_) – 44.765.

The measurement of these parameters enabled to classify the intensity of the ultramarathon in relation to the values recommended by the American College of Sports Medicine, as low (HR < 59% HR_max_ or V’O_2_^”^ < 49%), moderate (HR 60–79% HR_max_ or V’O_2_^”^ 50–74%), or high (HR > 80% HR_max_ or V’O_2_^”^ > 75%).

From these data, it was possible to obtain the relative V’O_2_ (ml/kg/min^–1^) and absolute V’O_2_^”^ (l/min).

#### Food Consumption During the Competition

During the competition, a list of consumed foods and their respective quantities was made, and delivered to the nutritionists at the end of the competition. The data obtained were transformed into energy values by means of the Dietbox software, to verify the total energy intake and consumption.

### Statistical Analysis

The results were expressed as means and corresponding standard deviations (SD). Statistical significance was considered if results showed a probability of occurrence of the null hypothesis less than 5% (*p* < 0.05). For comparison between before and after exercise, the paired *t*-test was used. Pearson’s correlation was also used to measure the degree of linear correlation between quantitative variables, by coefficient *r*-value. Graphs and statistical analyses were performed using the Prism 6.0 software.

## Results

Table 1 reports the age, anthropometric and hemodynamic profiles of the ultramarathon participants.

**TABLE 1:** Age, anthropometric variables measured at rest and hemodynamic variables measured at rest and during the cardiopulmonary exercise test of the ultramarathon participants (*n* = 7).

Variables	Mean ± SD
Age (Years)	44.71 ± 11.28
Weight (kg)	74.29 ± 11.25
Height (cm)	1.69 ± 0.06
BMI (kg/m^2^)	25.75 ± 3.20
Sum of skinfold measurements (mm)	120.40 ± 33.76
Body fat (%)	18.54 ± 4.35
HR_resting_ (bpm)	57.00 ± 10.28
SBP_resting_ (mm Hg)	129.01 ± 7.03
DBP_resting_ (mm Hg)	73.57 ± 6.55
Relative VO_2resting_ (ml/kg/min^–1^)	3.78 ± 0.31
HR_max_ of the test (bpm)	171.10 ± 9.75
Relative VO_2max_ of the test (ml/kg/min^–1^)	48.87 ± 4.78
Absolute VO_2max_ of the test (l/min)	3.61 ± 0.52
Threshold 1 (ml/kg/min^–1^)	36.58 ± 4.01
Threshold 2 (ml/kg/min^–1^)	42.12 ± 3.62

*BMI, body mass index; HR, heart rate; SBP, systolic blood pressure; DBP, diastolic blood pressure; V’O_2_”_max_, maximum oxygen consumption; max, maximum; SD, standard deviation; kg, kilograms; cm, centimeters; kg/m^2^, kilograms per square meter; mm, millimeters; bpm, beats per minute; mm Hg, millimeters of mercury; ml/kg/min^–1^, millimeters per kilogram per minute; l/min, liters per minute; min, minute; km, kilometers.*

The average duration of the competition (the average finishing time of the competitors) was 820.60 min (±117.00), during which the mean energy consumption was 2,209.72 kcal (±951.97) and energy expenditure was 7,837.16 kcal (±195.71), with 39.16 g/h ± 12.76 of carbohydrate consumption. In addition to these values during the competition, hemodynamic variables were collected. All variables collected during the competition are presented in Table 2.

**TABLE 2:** Participants variables measured during the ultramarathon (*n* = 7).

Variable	Mean ± SD
Duration (min)	820.60 ± 117.00
Energy consumption (kcal)	2209.72 ± 951.97
Energy expenditure (kcal)	7837.16 ± 195.71
HR _mean_ (bpm)	127.85 ± 12.02
HR _max_ of the test (%)	74.64 ± 4.54
Relative VO_2max_ of the test (%)	58.84 ± 7.70
Relative VO_2_ of the race (ml/kg/min^–1^)	28.75 ± 4.66
Absolute VO_2_ of the race (l/min)	2.10 ± 0.26
Total VO_2_ of the race (ltrs)	1733.00 ± 374.00

*HR, heart rate; V’O_2_”, oxygen consumption; max, maximum; SD, standard deviation; min, minute; kcal, kilocalorie; bpm, beats per minute; ml/kg/min^–1^, millimeters per kilogram per minute; l/min, liters per minute; ltrs, liters.*

We found a significant reduction (*p* < 0.05) of weight after (72.40 kg ± 9.78) the competition when compared to basal values (73.98 kg ± 10.25). Moreover, there was a very small decrease of body hydration after the competition (−2.11%). We also found an increase in RPE (*p* < 0.001) after the race, indicating the physical effort by the athletes (Table 3).

**TABLE 3:** Body weight and subjective perception of effort of athletes in the pre- and post-competition periods (*n* = 7).

Biomarker	Before Mean ± SD	After Mean ± SD	*P*-value
Body weight	73.98 ± 10.25	72.40 ± 9.78	0.0334
RPE	8.42 ± 1.27	15.86 ± 3.43	0.0006*

*RPE, rate of perceived exertion; SD, standard deviation.*

*The result was statistically significant (p < 0.05).

As shown in Table 4, the levels of glucose and cortisol were higher after the competition (*p* < 0.01), as well as creatinine and lactate levels (*p* < 0.001). In addition, there was a reduction of GFR (*p* < 0.001) and an increase (*p* < 0.001) of microalbuminuria after the competition.

**TABLE 4:** Blood and urine biomarkers of the athletes before and after the competition (*n* = 7).

Biomarker	Before		After		
	Mean ± SD	% of individuals with altered values (n)	Mean ± SD	% of individuals with altered values (n)	*P*-value
Glucose (mg/dL)	81.44 ± 12.14	0 (0)	95.42 ± 5.02	14.3 (1)^b^	0.0011*
Cortisol (μg/dL)	13.42 ± 1.56	0 (0)	22.43 ± 5.10	42.8 (3)^b^	0.0076*
Plasma creatinine (mg/dL)	0.71 ± 0.12	28.6 (2)^a^	1.57 ± 0.40	85.7 (6)^b^	0.0009*
Lactate (mg/dL)	9.9 ± 4.49	0 (0)	23.12 ± 8.23	57.1 (4)^b^	0.0007*
GFR (ml/min/1.73 m^2^)	145.66 ± 52.88	0 (0)	67.01 ± 28.48	42.8 (3)^a^	0.0004*
Microalbuminuria (mg/l)	2.01 ± 0.65	0 (0)	23.09 ± 9.23	57.1 (4)^b^	0.0009*

*GFR, glomerular filtration rate; SD, standard deviation; P-value, comparison between values before and after the competition (Student’s t-test); mg/dL, milligrams per deciliter; μg/dL, micrograms per deciliter; ml/min/1.73 m^2^, milligrams per minute of each 1.73 m^2^; mg/l, micrograms per liter.*

*The result was statistically significant (p < 0.05).

*^a^Blood and urine biomarkers values of the athletes before the competition (n = 7).*

*^b^Blood and urine biomarkers values of the athletes after the competition (n = 7).*

**FIGURE 2:**
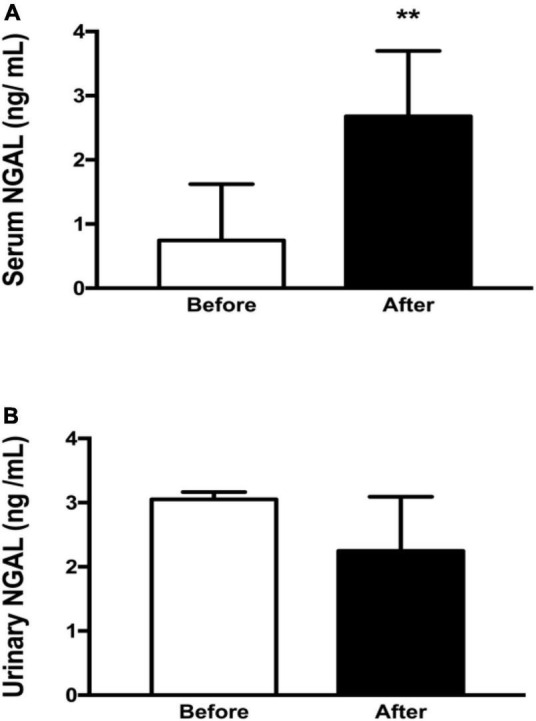
NGAL in blood and urine of the athletes before and after the competition (*n* = 7). **(A)** Serum NGAL values before and after ultramarathon. **(B)** Urinary NGAL values before and after ultramarathon. sNGAL, serum neutrophil gelatinase-associated lipocalin; uNGAL, urinary neutrophil gelatinase-associated lipocalin; ng/ml, nanograms per milliliter. The ** means that the result was statistically significant (*p* < 0.01).

The levels of sNGAL were higher after the competition (*p* < 0.01). However, no differences were observed in uNGAL levels (Figure 2).

**FIGURE 3:**
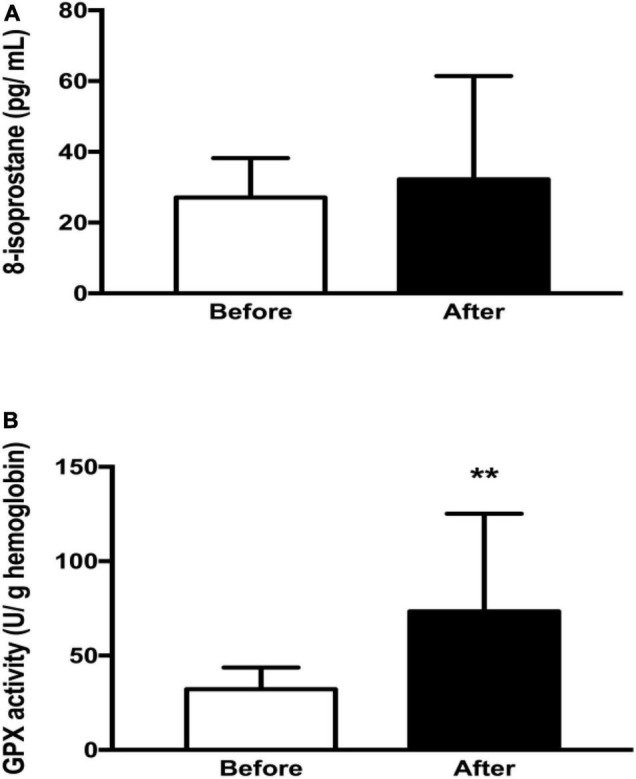
Oxidative stress biomarkers of the athletes before and after the competition (*n* = 7). **(A)** 8-isoprostane values before and after ultramarathon. **(B)** GPx activity before and after the ultramarathon. GPx, glutathione peroxidase; pg/ml^–1^, picogram per milliliter; u/g Hb, microgram per gram of hemoglobin. The ** means that the result was statistically significant (*p* < 0.01).

Regarding redox biomarkers, serum GPx activity was higher after the competition (*p* < 0.01), but there were no differences in serum 8-isoprostane levels (Figure 3).

In the pre-competition period, no significant correlations (*p* > 0.05) were found between NGAL levels, in blood and urine, and blood and urine biomarkers. There was also no significant correlation (*p* > 0.05) between the uNGAL and sNGAL values. In relation to the results obtained after competition, there were no significant correlations between sNGAL levels and plasma biomarkers, except for the serum creatinine level (*p* < 0.05). No differences were observed regarding the correlations between uNGAL and plasma and urinary biomarkers. Furthermore, no differences were observed in the correlation between the uNGAL and sNGAL levels (Table 5).

**TABLE 5:** Correlation matrix between sNGAL and uNGAL with blood and urine biomarkers pre and post competition (*n* = 7).

Variables Pre-competition	sNGAL		uNGAL	
	***r***	***p***	***r***	***p***
	
Lactate (mg/dL)	−0.60	0.14	−	−
Cortisol (μg/dL)	0.07	0.88	−	−
Creatinine (mg/dL)	0.10	0.82	0.37	0.46
8-isoprostane (pg/ml*^–^*^1^)	0.68	0.23	−0.80	0.06
GPx (u/g Hb)	0.46	0.08	0.05	0.92
GFR (ml/min/1.73 m^2^)	−	−	−0.55	0.25
Microalbuminuria (mg/L)	−	−	0.73	0.09
sNGAL (ng/ml)	−	−	−0.67	0.14

**Variables** **Post-competition**	**sNGAL**		**uNGAL**	
	
	***r***	***p***	***r***	***p***

Lactate (mg/dL)	−0.09	0.84	−	−
Cortisol (μg/dL)	0.57	0.23	−	−
Creatinine (mg/dL)	0.77	0.04*	0.10	0.83
8-isoprostane (pg/ml*^–^*^1^)	0.27	0.55	0.46	0.35
GPx (u/g Hb)	0.64	0.16	−0.31	0.54
GFR (ml/min/1.73 m^2^)	−	−	−0.16	0.75
Microalbuminuria (mg/L)	−	−	0.02	0.97
sNGAL (ng/ml)	−	−	−0.33	0.51

*GPx, glutathione peroxidase; GFR, glomerular filtration rate; sNGAL, serum; uNGAL, urinary neutrophil gelatinase-associated lipocalin; r, correlation coefficient; p, significance of the Pearson correlation test; mg/dL, milligrams per deciliter; μg/dL, micrograms per deciliter; pg/ml^–1^, pictograms per milliliter; u/g Hb, micrograms per gram of hemoglobin; ml/min/1.73 m^2^, milligrams per minute for each 1.73 m^2^; mg/L, milligrams per liter; ng/ml, nanograms per milliliter.*

*The result was statistically significant (p < 0.05).

## Discussion

The anthropometric profile of the athletes in our study was similar to that found in the literature (Rust et al., 2012; Hoffman, 2016). The mean value of V’O_2_”_max_ during the cardiopulmonary exercise test (48.87 ml/kg/min^–1^ ± 4.78) was similar to that found in a study of amateur ultramarathon runners whose profile was similar to our participants (50.75 ml/kg/min) (Sierra et al., 2016).

The values of thresholds 1 (36.58 ml/kg/min) and 2 (42.12 ml/kg/min) were, respectively, 74.85 and 86.18% of the value of V’O_2_”_max_ in relation to the test (48.87 ml/kg/min). It is known that the nearer these thresholds and V’O_2_”_max_ are to each other, the more intense exercise can be without developing metabolic acidosis and non-linear increase of respiratory difficulty, which, respectively, occur when thresholds 1 and 2 are exceeded (Brito et al., 2014; Taylor, 2014). These results are generally found in long-distance runners, indicating adaptations induced by exercise training.

Because of the long duration of ultramarathons, the runners try to maintain a low to moderate pace for most of the race, generally remaining below threshold 1 (Knechtle and Nikolaidis, 2018). In our sample, during the race, the athletes had relative V’O_2_” (28.75 ml/kg/min^–1^ ±4.66) below threshold 1 (36.58 ml/kg/min^–1^ ±4.01), enabling them to avoid excessive accumulation of lactate, and thus maintain a moderate pace, as shown by the mean values of% HR_max_ (74.64% ± 4.54) and%V’O_2_”_max_ (58.84% ± 7.70). However, there was a significant increase of plasma lactate levels (*p* < 0.001) after the competition (23.12 mg/dL ± 8.23), which was higher than the physiological reference value (4.5–19.8 mg/dL) (Diagnostic Labtest, 2019). It is plausible that this increase was due to the final sprint to the finish line. This assumption is based on studies indicating that, in 100 km ultramarathons, the lactate rises above the reference values only at the end of the competition (Fohrenback et al., 1987; Żoladz et al., 1993).

During the race, the athletes’ average carbohydrate intake (39.16 g/h ± 12.76) was within the range recommended by the International Society of Sports Nutrition, but was slightly lower than the average of participants in 100 km ultramarathons. And after the competition, the levels of glucose (95.42 mg/dL ± 5.02) and cortisol (22.43 μg/dL ± 5.10) remained, respectively, within their physiological reference ranges (60–99 mg/dL; 6.7–22.6 μg/dL) (Diagnostic Labtest, 2019).

In the present study we did not find differences in plasma 8-isoprostane levels before and after the race. However, we found one study showing higher levels of plasma 8-isoprostane after an 80 km ultramarathon (Pétibois, 2005). The difference between our results and those cited from the literature can be related to the different race strategies used by the athletes, which vary depending on the length and staging of the competition (Forman et al., 2015). In our study, we believe that fatigue limited the intensity at which the athletes performed the ultramarathon, which resulted in limited ROS production (Foulds et al., 2014; Ji et al., 2016). Furthermore, the significant increase in GPx activity after the race could also be associated with lower serum ROS levels (Liochev, 2013). Our results corroborate previous results, which provide evidence that prolonged exercise does not result in extreme oxidant-mediated cell damage and decreased antioxidant capacity in trained subjects (Foulds et al., 2014; Ji et al., 2016).

After the competition, we found a significant increase in creatinine levels (*p* < 0.001) and microalbuminuria (*p* < 0.001) levels, and a significant reduction in GFR (*p* < 0.001). Despite the reduction in GFR, the post-competition values (67.01 ml/min/1.73 m^2^ ± 28.48) were above the reference parameter (>60 ml/min/1.73 m^2^) (Diagnostic Labtest, 2019). It should be considered that creatinine measurement has limitations in its specificity and sensitivity (Racinais et al., 2015). Thus, the increase in creatinine (≥0.3 mg/dL), associated with the other results, indicates transient renal overload with greater predisposition to AKI (Gameiro et al., 2018). This assumption is supported by previous studies indicating that systemic and renal physiological changes tend to normalize after competition (Khwaja, 2012; Rojas-Valverde et al., 2021).

Furthermore, a previous study has found that immediately after an ultramarathon there was an increase in NGAL in blood and urine even before the increase in creatinine, reduction in GFR and onset of microalbuminuria (Schrezenmeier et al., 2017). However, in the present study, uNGAL levels were similar before and after the competition and were below the physiological reference limit (<20 ng/ml). On the other hand, sNGAL levels were higher after the competition (2.67 ng/ml) when compared to baseline values, but were also below the physiological reference limit (<70 ng/ml) (Pajek et al., 2014; Ho, 2015). It should be considered that the level of both sNGAL and uNGAL in AKI corresponds to 250 ng/ml (Nechemia-Arbel et al., 2008; Prowle et al., 2015). Recently, it has been proposed that subclinical elevations of NGAL, in plasma and urine, may indicate renal stress or mild injury with the possibility of subsequent impairment of function (Ho, 2015; Emlet et al., 2017). However, it has not yet been demonstrated in athletes (Rojas-Valverde et al., 2021).

We found only one study that has evaluated NGAL levels in plasma and urine immediately after an ultramarathon. The race in that study covered 60 km and the authors found a significant increase in NGAL (*p* < 0.001) afterward in plasma and urine, which exceeded the respective physiological reference limits (McCullough et al., 2011). A possible explanation for the difference between our results and those mentioned above is the intensity of exercise in the two competitions. Despite the lack of measurement of hemodynamic variables of the participants of the 60 km ultramarathon, we believe that the shorter distance of that race could have led the athletes to run faster on average, thus exerting more physical effort than our subjects. Furthermore, another research group has found significant positive correlations after a competition between sNGAL and creatinine, an indirect biomarker of damage (Decavele et al., 2011).

Studies claim that high blood and urine NGAL levels are harmful in the long term, since they are considered pro-inflammatory factors that promote progression to CKD (Ho, 2015; Hisamichi et al., 2017). The existence of a vicious circle between NGAL and inflammation has been suggested, in which NGAL, in blood and urine, aggravates inflammation, which in turn increases NGAL (Zhao et al., 2014; Eilenberg et al., 2016; Shao et al., 2016). For this reason, NGAL levels in urine and plasma are considered reliable biomarkers of inflammation. In addition, it is known that skeletal muscle damage related to long-distance running can be exacerbated by repetitive concentric-eccentric muscle contractions. Hence, increases in creatinine values, that are common during these types of events, may indicate a high rate of muscle damage due to the release of sarcoplasmic proteins into the bloodstream (Rojas-Valverde et al., 2019). Other researchers have proposed that high mechanical load is the main potentiating factor for AKI (Foulds et al., 2014; Hsu and Hsu, 2016; Shin et al., 2016; Rojas-Valverde et al., 2019). Thus, in our study, it is likely that a higher creatinine level after the race indicates that there has been muscle damage due to mechanical contractile stress, which possibly resulted in muscle inflammation and increased expression of sNGAL.

Furthermore, we did not observe a significant correlation between sNGAL and uNGAL in the period before or after the competition, which was also observed by McCullough et al. (2011). This suggests that sNGAL levels are more closely related to systemic inflammation, while uNGAL is related only to inflammation in the kidneys. This hypothesis is based on the fact that sNGAL is expressed in kidneys, endothelium, liver, lungs, large intestine, and innate immune system, while uNGAL is produced exclusively in the kidneys (Liu et al., 2015; Eilenberg et al., 2016). Moreover, we observed a significant correlation between creatinine and sNGAL. The higher creatinine levels after the race indicated there was muscle damage due to mechanical\contractile stress, which possibly resulted in muscle inflammation and thus increased expression of sNGAL (Foulds et al., 2014; Shin et al., 2016).

The seven participants in this study had age between 33 and 55 years old, so it is important to discuss the influence of age in our analysis as well as the statistical power of our study. The aging process is related to the loss of muscle mass, lower physical activity levels, and reduced food intake, leading the elderly people to have lower creatinine generation (Hirsch et al., 2007; Murray et al., 2008). Although the participants were aged between 33 and 55 years, all of them were athletes with good health conditions. The incidence of AKI is more common over 60 years of age due to a decline in renal function and multiple comorbidities, among other factors (Chao et al., 2015). We could not find in the literature any study showing the influence of age on the levels of sNGAL or uNGAL. This is an important topic that requires future studies. Moreover, the comparison between young and older athletes in relation to sNGAL and uNGAL levels after exercise is also a research topic that needs attention. The statistical power calculated by the STATA program was 98.67%, considering the mean and standard deviation values of sNGAL (the most important variable in the study), before (mean = 0.74; *SD* = 0.88) and after (mean = 2.68; *SD* = 1.02) the ultramarathon, together with the α value of 5%. Thus, our sample of seven participants was sufficient to detect statistical differences between the periods, avoiding type II errors (false-negative errors).

We did not find a significant correlation between the levels of uNGAL and the urinary biomarkers, either before or after the race. This result was also observed in two studies that evaluated subjects after a 60 km ultramarathon or cyclists analyzed 48 h after an intense exercise (McCullough et al., 2011; Julio et al., 2018).

Neither sNGAL nor uNGAL showed significant correlation (*p* > 0.05) with redox biomarkers (8-isoprostane and GPx), both before and after the competition. This suggests that NGAL levels are probably not exclusively induced by ROS and reinforces the evidence that NGAL is more sensitive to an inflammatory process resulting from muscle injury. This suggestion is based on studies that have found that NGAL can be positively regulated by oxidative stress, inflammation, apoptosis, and fibrosis (Liu et al., 2015; Eilenberg et al., 2016). Moreover, since 8-isoprostane was not altered in our study, it is possible that the amount of ROS production was not sufficient to induce an inflammatory process and consequently AKI (Liochev, 2013; Tanaka, 2017).

## Conclusion

There was no significant correlation between oxidative stress biomarkers and NGAL in serum and urine. This suggests that NGAL is more sensitive to the inflammatory process than to ROS production, as thee was a significant correlation only with creatinine. Thus, the results found in the present study suggest that NGAL is a possible early biomarker of inflammatory processes and possibly of AKI.

The main limitation of this study is the uncertainty about the source that led to the increase of sNGAL. Thus, further research is needed to investigate the systemic response, in particular the renal response, for sequential periods after ultramarathons.

## Data Availability Statement

The raw data supporting the conclusions of this article will be made available by the authors, without undue reservation.

## Ethics Statement

The studies involving human participants were reviewed and approved by the Ethics Committee of Ceará State University (CEP/UECE) under number 3.454.557. The patients/participants provided their written informed consent to participate in this study.

## Author Contributions

ACCL and GFN participated in all data collections and wrote the manuscript. ALLC and RSM helped to carry out the anthropometric collections, PSE and body weight. JCCNF and EFD performed the NGAL, blood and urine tests and statistical analyses. FHMP performed the cardiopulmonary exercise test. ACO performed the biochemical analyses and analysis of food consumption. VMC assisted in carrying out the biochemical analyses. RSF helped to wrote the manuscript. DPC participated in the design and coordination of the study. All authors read and approved the final version of the manuscript and agreed with the authors’ order of presentation.

## Conflict of Interest

The authors declare that the research was conducted in the absence of any commercial or financial relationships that could be construed as a potential conflict of interest.

## Publisher’s Note

All claims expressed in this article are solely those of the authors and do not necessarily represent those of their affiliated organizations, or those of the publisher, the editors and the reviewers. Any product that may be evaluated in this article, or claim that may be made by its manufacturer, is not guaranteed or endorsed by the publisher.
